# The Knowns Unknowns: Exploring the Homologous Recombination Repair Pathway in *Toxoplasma gondii*

**DOI:** 10.3389/fmicb.2016.00627

**Published:** 2016-05-03

**Authors:** Ignacio M. Fenoy, Silvina S. Bogado, Susana M. Contreras, Vanesa Gottifredi, Sergio O. Angel

**Affiliations:** ^1^Laboratorio de Parasitología Molecular, IIB-INTECH, CONICET-UNSAMChascomús, Argentina; ^2^Cell Cycle Genomic Instability Laboratory, Fundación Instituto Leloir, IIBBA-CONICETChascomús, Argentina

**Keywords:** *Toxoplasma*, DNA damage, homologous recombination repair, chromatin, fork collapse, double strand break

## Abstract

*Toxoplasma gondii* is an apicomplexan parasite of medical and veterinary importance which causes toxoplasmosis in humans. Great effort is currently being devoted toward the identification of novel drugs capable of targeting such illness. In this context, we believe that the thorough understanding of the life cycle of this model parasite will facilitate the identification of new druggable targets in *T. gondii*. It is important to exploit the available knowledge of pathways which could modulate the sensitivity of the parasite to DNA damaging agents. The homologous recombination repair (HRR) pathway may be of particular interest in this regard as its inactivation sensitizes other cellular models such as human cancer to targeted therapy. Herein we discuss the information available on *T. gondii*'s HRR pathway from the perspective of its conservation with respect to yeast and humans. Special attention was devoted to BRCT domain-containing and end-resection associated proteins in *T. gondii* as in other experimental models such proteins have crucial roles in early/late steps or HRR and in the pathway choice for double strand break resolution. We conclude that *T. gondii* HRR pathway is a source of several lines of investigation that allow to to comprehend the extent of diversification of HRR in *T. gondii*. Such an effort will serve to determine if HRR could represent a potential targer for the treatment of toxoplasmosis.

## Introduction

The protozoan parasite *Toxoplasma gondii* is a medical and veterinary relevant pathogen (Tenter et al., 2000; Pfaff et al., 2014). *Toxoplasma* belongs to *phylum Apicomplexa* among other important human and veterinary parasites such as *Plasmodium* spp., *Cryptosporidium* spp., *Eimeria* spp. Albeit the toxoplasmic infection is usually asymptomatic, severe complications, and even death might occur as a result of a congenital infection or in immunocompromised individuals (e.g., AIDS, transplantation). Congenital toxoplasmosis causes several types of neurological defects, chorioretinitis and in some cases even abortion (Cortés et al., 2012; Moncada and Montoya, 2012; Torgerson and Mastroiacovo, 2013). In immunocompromised patients, the reactivation of the infection may trigger further complications including neurological defects, and encephalitis (Yan et al., 2013).

*T. gondii* is an intracellular obligated protozoan parasite with a life cycle that includes sexual and asexual stages. Asexual replication occurs in a wide variety of intermediate host species and tissues and is characterized by two stages: the rapidly growing “tachyzoites” which is sensitive to the immune system of the host and several drugs, and the slowly dividing encysted “bradyzoites” which evades both the host immune response and currently available anti-*Toxoplasma* drugs (Dubey, 1998; Weiss and Kim, 2000). Besides, anti-folate treatment is only effective against the tachyzoite stage, but is toxic in that it causes bone marrow depression; moreover, many patients are allergic to the sulfa drug component (Baatz et al., 2006; Cortés et al., 2012). The pathogenicity of toxoplasmosis has been associated to multiple cycles of host cell invasion, intracellular division of the parasite and release from host cells. *T. gondii* amplification takes place in any nucleated cell within a parasitophorous vacuole generated by an internal budding process known as endodiogeny (Gubbels et al., 2008; Francia and Striepen, 2014).

While many molecular pathways including cell cycle and cell duplication were thoroughly characterized in the parasite, the molecular signals ruling DNA replication in *T. gondii* are yet poorly characterized. Notably, after host cell invasion, the tachyzoite replicates with a doubling time of 5–9 h (Radke et al., 2001). We have recently proposed that such fast and uninterrupted rounds of DNA replication during the tachyzoite stage might trigger replication stress. In fact, we have evidenced a striking increase in the levels of a *bona-fide* replication-stress marker, the phosphorylation at Ser132 of γH2A.X, in *T. gondii* tachzyoite (Dalmasso et al., 2009). Albeit other replication-associated defects may also trigger γH2A.X activation, the classical interpretation of γH2A.X accumulation is the generation of double strand break (DSB) (Redon et al., 2002; Tu et al., 2013; Turinetto and Giachino, 2015). DSBs are extremely genotoxic DNA lesions capable of impairing central DNA process such as DNA transcription, replication, and segregation. Given that DSBs can be repaired by more than one mechanism, DSBs accumulated during the DNA replication of tachyzoite most likely require a precise choice of DNA repair pathway. A failure or a delay in the repair of DSBs may trigger cell death due to the accumulation of genomic and chromosomic rearrangements as has been showed in cancer cells (Prakash et al., 2015).

If DSBs accumulate during the DNA replication of tachyzoite, it is important to discuss the DNA repair pathways available for the repair of DSBs in *T. gondii*. In Eukaryotes, two well-characterized pathways are in charge of DSB Repair: Homologous Recombination repair (HRR) and Non-Homologous End Joining (NHEJ). While it is broadly accepted that HRR is error-free and NHEJ is error-prone, new evidence suggests that, at least, under certain cirscutances, HRR can also represent an error-prone mechanism and NHEJ can be very precise depending on the structure of the DNA ends (Betermier et al., 2014; Guirouilh-Barbat et al., 2014).

Intriguingly, while most DNA repairs pathways are conserved in *T. gondii*, recently reviewed in Smolarz et al. (2014), differences in the HRR cascade have been reported in different organisms (Smith, 2012; Blackwood et al., 2013; Daley et al., 2013; Yoshiyama et al., 2013). Suchdiversification indicates the existence of a window of opportunity for the identification of specific HRR components in *T.gondii*. If available, such factors could represent attractive candidates for the development of drug against toxoplasmosis. Hence, herein we analyze the extent of conservation between the HRR components of *T. gondii* and their yeast and human counterparts.

## The homologous recombination in *T. gondii*

HRR is preferentially an error-free mechanism which represents the preferred pathway chosen in eukaryotes for the repair of DSBs during the late S/G2-phases of cell cycle. This mechanism has been extensively studied in both yeast and higher eukaryotes (Daley et al., 2013; Jasin and Rothstein, 2013). The restriction of HRR to S/G2 phases is linked to the requirement of homologous sequences as a template for DNA repair (Sancar et al., 2004). Typical substrates for HRR include: (a) direct double-ended DSBs generated by genotoxic agents such as γ-irradiation and X-rays, (b) inter-strand crosslinks generated after exposure to genotoxins such as mitomycin C (MMC), and (c) one-ended DSBs generated after fork collapse resulting from persistent stalling at bulky adduct or at naturally-occurring replication barriers. The resolution of direct DSBs by an HRR subpathway may or may not involve crossing over. One-ended DSBs are expected to be resolved by another HRR sub-pathway involving long range D-loop migration (break-induced repair) (Carr and Lambert, 2013; Malkova and Ira, 2013). If homologous sequences are not available, for example during G1, DSBs are repaired by NHEJ, a pathway that prompts rapid fusion between the ends of double-ended DSBs. In contrast, NHEJ is disfavored during S phase since its activation at one-ended DSBs can jeopardize genomic instability by fusing non-homologous chromosomes. Hence, while NHEJ can function along the cell cycle (Shibata and Jeggo, 2014), NHEJ is the pathway chosen for the repair of DSBs in G1 and HRR is preferentially activated at collapsed replication forks during S and G2 phases (Johnson and Jasin, 2000; Sancar et al., 2004; Blackwood et al., 2013).

Effectors of the HRR and NHEJ pathways were identified in *T. gondii* (Smolarz et al., 2014). When attempting to establish the hierarchy between both pathways in the parasite, surprising results were obtained. The inoculation of linear plasmid in tachyzoites robustly activates the NHEJ pathway, while gene replacement by HRR was rarely detected (Fox et al., 2009). Notably, these results suggested that, in contrast to yeast and humans, the NHEJ pathway is the pathway preferentially used by *T. gondii*. It should however be mentioned that HRR can efficiently be activated in *T. gondii* when NHEJ factors Ku70/Ku80 are eliminated by means of deletion of the Ku80. In such scenario efficient HRR-dependent integration rate at correct locus of different plasmid constructions were observed (Fox et al., 2009; Huynh and Carruthers, 2009). Moreover, when focusing on events such as crossing over, a high efficiency of activation was observed, hence indicating active HRR during sporozoite development (Khan et al., 2014). Together, these evidences demonstrate that *T. gondii* has an intact and functional HRR molecular pathway.

## The homologous recombination basic machinery is conserved in *T. gondii*

**Figure 1:**
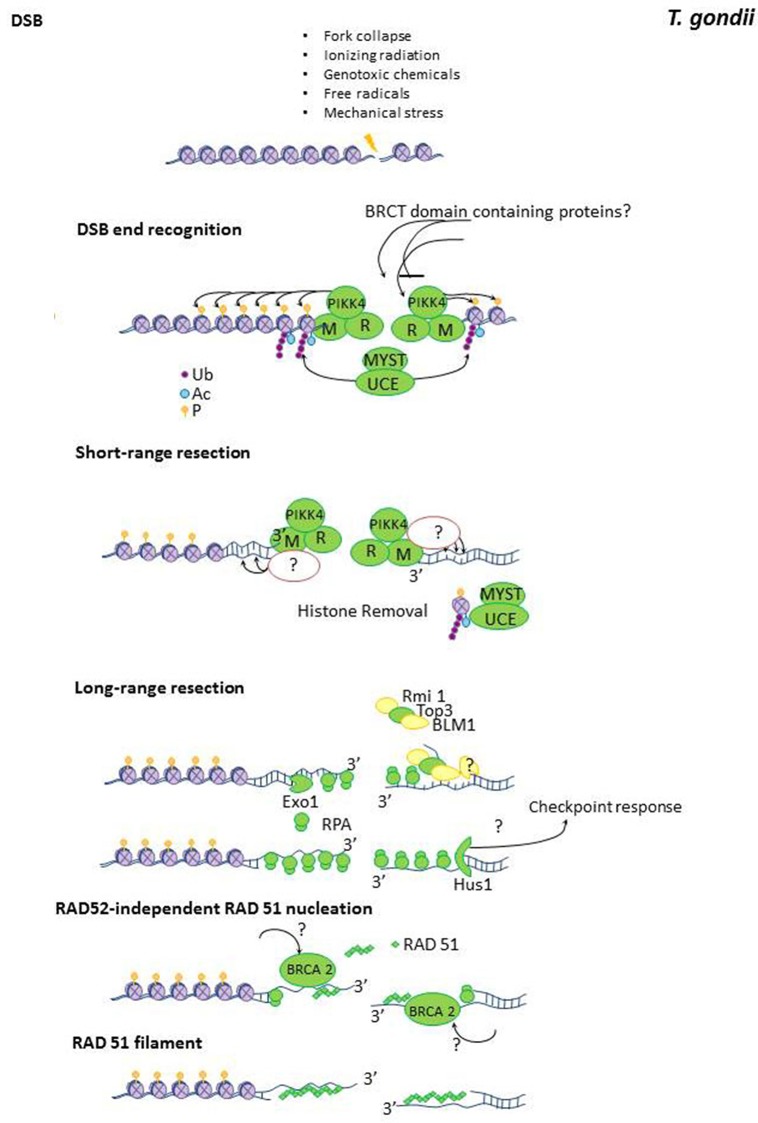
**Homologous recombination repair in *T. gondii***. Potential components of the HRR pathway in *T. gondii*. The pathway was build by taking the HRR components described in mammals, in yeast as references and using the information regarding the putative HRR protein retrieved from www.toxodb.org. TGME49_258480, TGME49_239790, and TGME49_237480 are putative BRCT domain containing proteins. PIKK4 is the putative ATM/Tel1 kinase TGME49_248530. UCE is a ubiquitin conjugate enzyme. High levels of conservation between *T. gondii* and humans/yeast protein domains are indicated in green. Proteins which are not detected by annotation but have compatible features with the respective protein are shown as yellow shapes.

The HRR pathway is activated after DSB recognition by DNA damage sensors (e.g., γH2A.X), and signal transducers (e.g., ATM/Tel1 PIKK4 kinase). The commitment of DSBs to HRR resolution is achieved by mediators/adaptors (e.g., BRCA1 in mammals) and effectors (e.g., Mre11, RAD50, Nbs1/Xrs2 complex; Prakash et al., 2015). HRR core components include many DNA damage repair (DDR) protein (e.g., RAD51, BRCA2, RAD52) that regulate homology search and other downstream events(Jasin and Rothstein, 2013). Herein we evaluate whether mammalian and yeast factors are present in *T. gondii* by Gene Text Search at Toxodb database (Table S1, Figures S1, S2, and **Figure 3**). Table S1 also contains putative HRR counterparts from *Plasmodium falciparum*, another apicomplexan parasite. We found 39 putative HRR components in *T. gondii* (Table S1). In addition, we have attempted to infer whether the conserved HRR factors retrieved in *T. gondii* are sufficient to support full HRR activation when establishing a direct comparison with the essential components of the HRR cascade in yeast and humans (Figure S2). As a result we have generated a putative basic model of *T. gondii* HRR (Figure 1). The more relevant HRR proteins found in *T. gondii* are listed in Table 1.

**Table 1:** **DNA damage checkpoint and homologous recombinantion putative proteins in *T. gondii***.

**Mammalian**	**Yeast**	***T. gondii***	**Toxodb annotation**
53BP1 BRCA1 MDC1	RAD9	TGME49_239790 TGME49_237480 TGME49_258480	BRCA1 C Terminus domain-containing protein BRCA1 C Terminus domain-containing protein Hypothetical protein^*^
Abraxas	ND	ND	ND
ATM	Tel1	TGME49_248530	FATC domain containing protein
Bard1	ND	ND	ND
BLM^+^	Sgs1	ND	ND
BRCA2	ND	TGME49_243265	Protamine P1 protein^*^
BRCC36	ND	TGME49_308590	Mov34/MPN/PAD-1 family protein
CK2alpha	CKA2	TGME49_263070	CMGC kinase, CK2 family
CK2beta	CKB1	TGME49_272400	Casein kinase ii regulatory subunit protein
CtIP	Sae2	ND	ND
DNA2	Dna2	TGME49_269740	R3H domain-containing protein^*^
DNAPd	DNAPd	TGME49_258030	DNA polymerased
DNAPh	ND	TGME49_237830	DNA polymerase I
EME1	MMS4	ND	ND
ERCC1	Rad10	TGME49_249330	Rad10 subfamily protein
ERCC4	Rad1	TGME49_305310	ERCC4 domain-containing protein
EXO1	Exo1	TGME49_233090	XPG N-terminal domain-containing protein
FANCD2	ND	ND	ND
FANCF	ND	ND	ND
FANCM	Mph1	ND	ND
GEN1	Yen1	TGME49_251620	Flap structure-specific endonuclease 1
MRE11	Mre11	TGME49_278060	Mre11
MUS81/ERCC4	Mus81	TGME49_261610	Hypotetical protein^*^
Nbs1	Xrs2	ND	ND
H2A.X	HTA2	TGME49_261580	H2A.X
Hop2	Hop2	ND	ND
PALB2/FANCN	ND	ND	ND
PCNA	PCNA	TGME49_247460	Proliferating cell nuclear antigen 1
		TGME49_320110	Proliferating cell nuclear antigen 2
RAD50	Rad50	TGME49_257180	RecF/RecN/SMC N terminal domain-containing protein^*^
RAD51	Rad51	TGME49_272900^**^	DNA repair protein RAD51
RAD51AP1	ND	ND	ND
RAD52	Rad52	ND	ND
RAD54		TGME49_232450	SWI2/SNF2-containing protein RAD54
RAP80	ND	ND	ND
RMI1^+^	Rmi1	ND	ND
RMI2^+^	ND	ND	ND
RNF168	Rad18	ND	ND
RNF8	Dma2	ND	ND
RPA1A	RFA1	TGME49_236080	Replication factor a protein 1
RPA2	RFA2	ND	ND
RPA3	RFA3	TGME49_214480	Replication factor a protein 3
GIY-YIG_SLX1	GIY-YIG_SLX1	TGME49_212170	GIY-YIG catalytic domain-containing protein
SLX4 (FANCP)	Slx4	TGME49_277540	Hypotetical protein^*^
SMC1	Smc1	TGME49_288700	RecF/RecN/SMC N terminal domain-containing protein
SMC3	Smc3	TGME49_297800	RecF/RecN/SMC N terminal domain-containing protein
SPO11	Spo11	ND	ND
TIP60	Esa1	TGME49_207080	Histone lysine acetyltransferase MYST-B
TOPOIII	Top3	TGME49_264450	DNA topoisomerase III beta-1
UBC13^++^	Ubc13	ND	ND
WRN	ND	TGME49_306080	ATP-dependent DNA helicase, RecQ family protein
XRCC2	ND	ND	ND
XRCC3	ND	ND	ND
ChK1	Chk1	ND	ND
ChK2	Rad53	TGME49_207820	Cell-cycle-associated protein kinase MAPK^*^
p53	ND	ND	ND
CDC25	YCH1	ND	ND

* *Comment at toxodb (see respective geneID)*.

** *AFN55127*.

+ *T. gondii database has several RecQ family proteins*.

++ *T. gondii database has several Ubiquitin-conjugating enzyme E2 family proteins*.

### Toxoplasma

HRR will be discussed below and will be organized accordingly to the following HRR stages: (A) DSB recognition, (B) end-resection and generation of protruding ends for homologous search, (C) strand invasion, (D) homologous DNA synthesis, and (E) resolution of DNA- repair intermediates.

### DSB recognition

The proteins in charge of DSB recognition are well-conserved in all three kingdoms. In bacteria, DSBs are recognized by SbcD and SbcC while in *Archaea* and Eukaryota these components are known as Mre11 and RAD50, respectively (Blackwood et al., 2013). Yeast and vertebrates have an additional highly divergent protein, Xrs2 (yeast) and Nbs1 (higher eukaryotes), which along with Mre11 and RAD50 form the MRX/N complex. From *T. gondii* database analysis it could be inferred that the Mre11 and RAD50 proteins are present, while Nbs1 was not detected in the database (Table 1 and Table S1). Recently, a functional plasmodial Mre11(PF3D7_0107800, Tables S1), similar to putative *T. gondii* Mre11, was identified (Badugu et al., 2015). The lack of Nbs1/Xrs2 is unexpected since Nbs1/Xsr2 is required for optimal activation of the checkpoint kinase ATM which is required for the arrest of the cell cycle and to trigger DNA damage-induced apoptosis (Difilippantonio and Nussenzweig, 2007). Nbs1 senses the conformation of Mre11 dimer, which is in turn influenced by RAD50-ATP state, promoting the activation of Mre11 (Lafrance-Vanasse et al., 2015). Nbs1 possesses a forkhead associated (FHA) domain and two breast cancer-associated 1C terminus (BRCT) domains known to bind phosphoproteins such as CtIP facilitating its recruitment at DSB (Williams et al., 2009). Moreover, Nbs1 also interacts with ATM through its C-terminal FXF/Y motif promoting its activation (You et al., 2005). We speculate that Nbs1 is not annotated in *T. gondii* genome database possibly due to its tendency to diverge. However, based on the above-mentioned data we believe that the MR complex, in charge of DSB recognition is mainly conserved in *T. gondii* (Figure 1).

### End-resection

In order to generate protruding ssDNA ends with invasion capacity, DSBs need to be extensively processed after MRN loading. Central enzymes capable of achieving such processing are the single strand 3′-5′ exonuclease and endonuclease Mre11 and the endonuclease CtIP [CtBP (C-terminal-binding protein)-interacting protein] (Sae2 in yeast). After an initial cleavage by Mre11, a second end-resection in eukaryotes depends mainly upon the Exo1 5′-3′ exonuclease which exerts long end resection forming the protruding DNA ends required for invasion and homologous search. An alternative pathway to end resection involves the Dna2 exonuclease and the BLM helicase (Figures S1, S2). Although CtIP (Sae2) is not identified in *T. gondii* database (there is a Sae2/CtIP annotated protein [TGVEG_252280] in toxodb but to our knowlege with no BLASTP evidence that support it.), a conserved Mre11 (see above) and a putative Exo1 exonuclease (Table 1) are present. Therefore, the end-resection stage of HRR is potentially conserved in this organism (Figure 1).

### Strand-invasion

In this phase, the protruding ssDNA is coated with a factor known as RecA in bacteria, RAD51 in eukaryotes or RadA in *Archae*. RAD51 facilitates strand invasion and homology search (Jasin and Rothstein, 2013). To promote RAD51 loading, factors known as mediators facilitate the displacement of the ssDNA coating factor, RPA. In eukaryotes, RAD51 is recruited by RAD52 or BRCA2 (Liu and Heyer, 2011). RAD51-coated ssDNA actively searches for homologus DNA, an event which is facilitated by increased chromosome moving (ICM) promoted by protein such as Rad9, RAD51, RAD54, Mec1/ATR, among others (Mine-Hattab and Rothstein, 2013) The analysis of *T. gondii* database revealed a putative sequence for RAD51 and BRCA2 but not RAD52 (Table 1). In fact, TgRad51 has been characterized by Achanta et al. (2012). When the authors compared it to a yeast cell model, they concluded that TgRad51 is less efficient in gene targeting and gene conversion than yeast Rad51. We speculate that a slight defect in this particular event may support the puzzling preponderance of NHEJ in *T.gondii* which has been discussed in previous sections. In fact, in the next section we will present the multiple levels of cross-regulation between HRR and NHEJ mediators and their major influence in the DSBs repair pathway choice.

### Homologous DNA synthesis and resolution of DNA- repair intermediates

**Figure 2:**
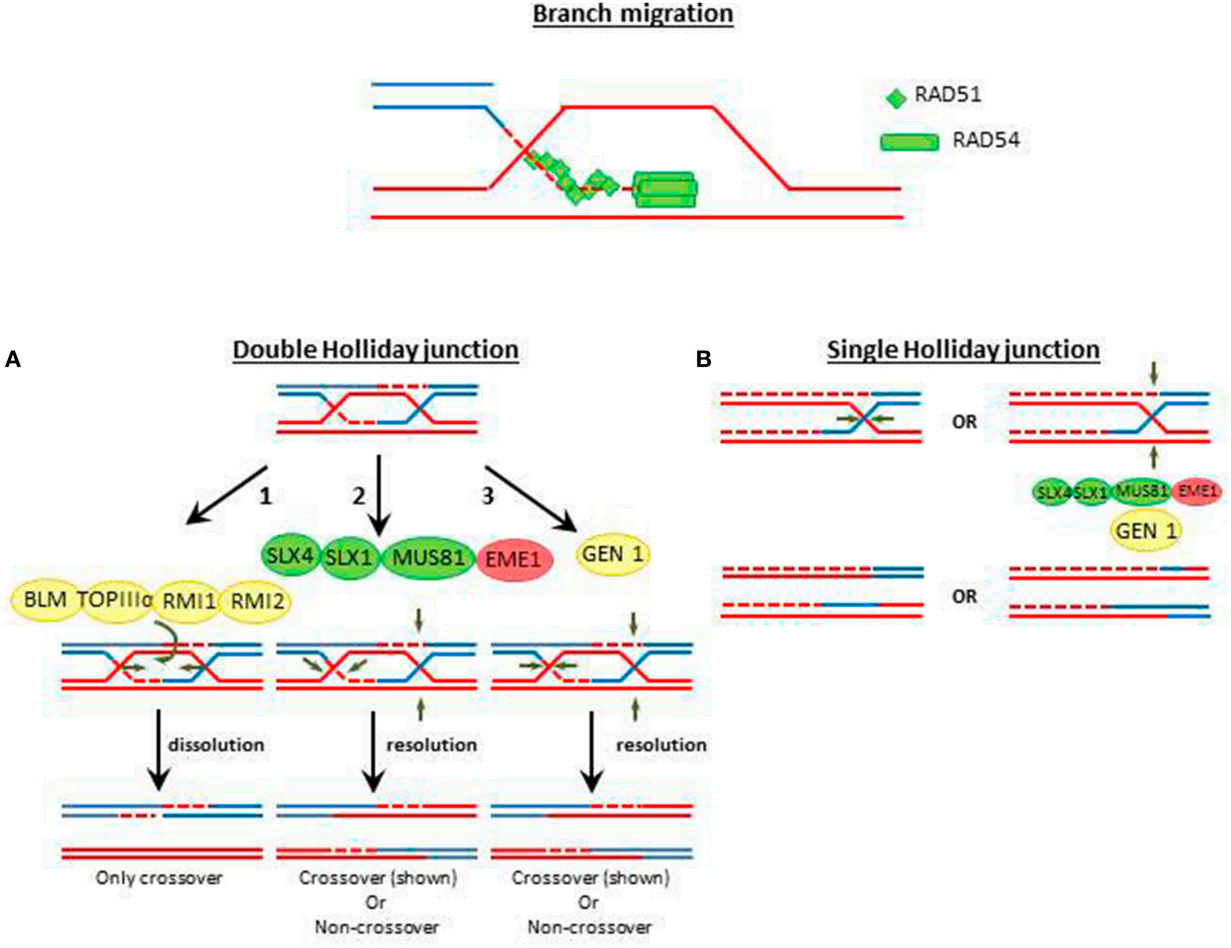
**Holliday junction resolution pathways**. Double Holliday junction dissolution is a conserved mechanism by which crossover is prevented and noncrossover HRR events are facilitated (Sarbajna and West, 2014). The Double Holliday junction (DHJ) dissolution is promoted by the BLM helicase, the TopoIIIα topoisomerase and the RMI1, which influencing the dynamics of TopIIIα (Bocquet et al., 2014). Double Holliday junctions are resolved by two mechanisms. **(A)** The nuclease complex MUS81, EME1, SLX1, and SLX4 generate asymmetric cleavage at two positions in the DHJ. **(B)** The GEN1 resolvase introduces two symmetrical nicks at equivalent positions of the DHJ. In both scenarios non-crossover o crossover resolutions are possible. Single Holliday junctions are intermediates of meiotic recombination. Proteins with high level of conservation are colored in green. Proteins which are not detected by annotation but have compatible features with the respective protein are shown as yellow shapes and factors wich have not been yet identified in in *T. gondii* are colored in red.

From mammals to yeast, once RAD51 bounds to ssDNA it generates a contiguous helical nucleoprotein filament, which searches for an intact homologous dsDNA template (Figure 2 and Figure S1). When the homologous region is found, RAD51 promotes the exchange of DNA strands leading to the formation of joint molecules and D-loops (Mehta and Haber, 2014). RAD54, a member of the Snf2-family of SF2 helicases also binds to RAD51 (Figure 1). Instead of taking part in the separation of the DNA duplex, RAD54 acts as a motor protein that translocates on duplex DNA and remodels specific protein–duplex DNA complexes (Pazin and Kadonaga, 1997; Ceballos and Heyer, 2011). The homology between RAD54 and Snf2/Swi2 further supports a role of RAD54 in chromatin relaxation during HRR, which could facilitate many HRR events such as Rad51 filament assembly, homology search, DNA strand invasion, or even later HRR stages (Ceballos and Heyer, 2011). In fact, RAD54 is crucial to promote branch migration (Mazin et al., 2010) when the DNA polymerase polη extends DNA from D loop recombination intermediates, using an invading strand as a primer, (McIlwraith et al., 2005), that generate a Holliday junction (HJ).

Nucleases in charge of HJ resolution are the ERCC1-XPF/SLX1/SLX4 and the Mus81-EME1/Mms4 complexes (Cejka, 2015). A third complex which may also resolve HJ when SLX4 is absent is the BLM/GEN1 nuclease (Garner et al., 2013). MUS81-EME1/Mms4 are essential components of HJ resolvase (Boddy et al., 2001) and seems to be the preferred nuclease in charge of the processing of crossover events while GEN1 seems to work as a backup pathway (Garner et al., 2013). Such hierarchy is also influenced by the cell cyle. During unperturbed duplication, different kinases (e.g., Mitosis phase CDKs) and phosphatases restrict the activity of MUS81-EME1 and GEN1 to different cell cycle phases. As a consequence of such regulation MUS82-EME/Mms4 are active during pro-metaphase and metaphase whereas GEN1/Yen1 are active during metaphase and anaphase (Matos and West, 2014).

*T. gondii* possess a conserved machinery responsible of HJ resolution (Table 1 and Figure 2). Still, to this date none of their components were experimentally characterized. Based on *T. gondii* annotation, RAD54, and TOPOIIIα are potentially expressed in the parasite (Table 1). MUS81 and SLX1 nucleases and the SLX4 scafolding factor are present in *T. gondii*. The same analysis provided modest evidence supporting the presence of the BLM helicase, the GEN1 nuclease and the RecQ-mediated genome instability protein 1 (RMI1). EME1/Mms4 was not found in *T. gondii* database (Table S1). Moreover, *T. gondii* database only retrieved two putative ERCC4 domain containing proteins, one resembling MUS81 and another displaying similatities with the RAD1/ERCC4-XPF endonuclease. The absence of EME1/Mms4 may indicate the existence of divergent proteins which were not yet identified. Alternatively, it is also possible that most crossover events in *T. gondii* relay exclusively on the GEN1 pathway. Further studies should reveal the mechanism supporting crossover and HJ resolution in the parasite.

The sexual cycle of *T. gondii* occurs in felines which serve as the definitive host and shed infectious oocysts in their feces. Meiosis events take place after oocyst sheed to generate haploid sporozoites. In a recent study, the mixture of Me49 and VAND strains in cats revealed both conventional and double-crossover HRR events (Khan et al., 2014). Moreover, Khan et al. (2014) has reported elevated frequency of small double-crossover events (less 1000 bp). Interestingly, double crossover events within the 1000 bp are classified as gene conversion, a mechanism associated with HRR-dependent resolution of DSB in other systems (Haber et al., 2004; Chen et al., 2007).

Collectively, the examination of the different HRR steps in *T. gondii* indicates that the basic HRR machinery is conserved, with the unanticipated exception of few but very important players including Nbs1/Xrs2, CtIP, RAD52 and EME1/Mms4. It is however important to mention that the evidences of a functional HRR pathway in *T. gondii* is solid (as it will be discussed in the next section). Thereafter we propose that the “missing” HRR components may have diverged to the point of not being recognized by data mining. Alternatively they may have been replaced by functional paralogs. In both scenarios, the identification of those central HRR components may serve as a tool to boost the rational design of drugs that may specifically impair HRR in the parasite.

## The DSB repair pathway choice in *T. gondii*

**Figure 3:**
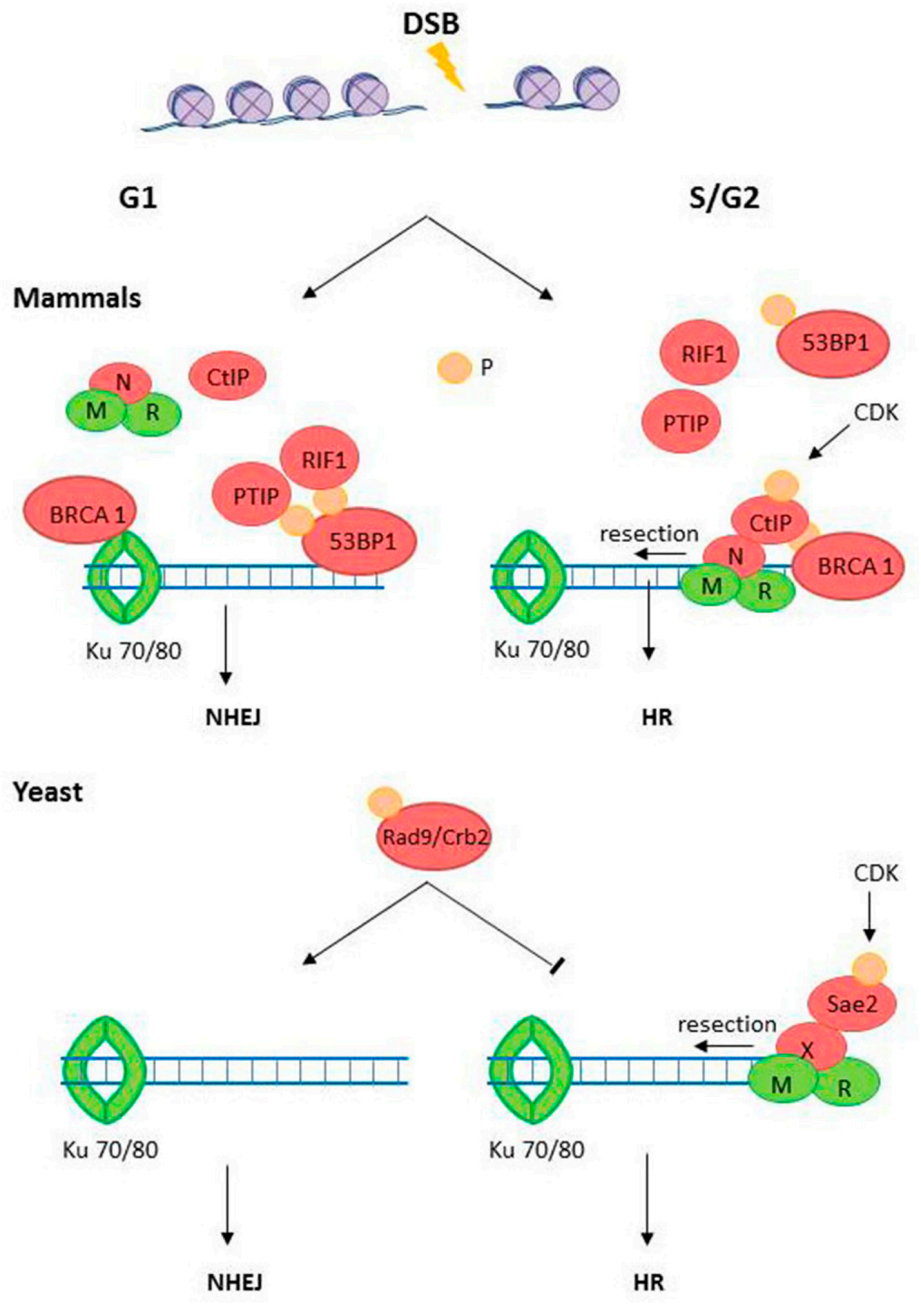
**The DSB pathway choice**. DSB could be repaired either by homologous recombination repair (HRR) during S/G2/M phases or by non-homologous end-joining (NHEJ) during G1 phase. The endonuclease CtIP (Sae3 in yeast) is a target of cyclin dependent kinases (CDKs) during G2/S-phase and is recruited to the DSB by the MRN (MRX in yeast) complex. In mammals, CtIP recruits BRCA1 to DSBs, a BRCT-containing factor that releases the NHEJ factor 53BP1 from the DBS site. 53BP1, in opposition to BRCA1, inhibits end resection, and promotes NHEJ activation. In yeast, the BRCT domain containing protein Rad9/Crb2 has shown similar role than 53BP1 in mammals. Phosphorylated CtIP also promotes end resection which evicts Ku70/Ku80 and commits the DSB to HRR. Proteins with high level of domain conservation are represented as green shapes, Proteins that are not present in *T. gondii* are represented as red shapes.

The current understanding of the DSB repair pathway choice in mammals is summarized in Figure 3 (Ceccaldi et al., 2016). In the case of mammals, the cell cycle majorly influences the DSB pathway choice. While HRR is the preferent choice during S/G2, NHEJ is the best option during the G1 phase(Sancar et al., 2004; Kass and Jasin, 2010). Such a strong influence of the cell cycle is accepted to depend on the availabity of intact sister chromatid during late S and G2 phases of the cell cycle (Kass and Jasin, 2010). Therefore, It's crucial to understand why NHEJ is dominant over HRR in *T. gondii*. On one hand, *T. gondii* tachyzoite has a cell cycle with a long G1 and no G2 phase (Radke et al., 2001). On the other hand, as we discuss bellow, the diversification in the molecules in charge of the commitment of a DSB to a given resolution pathway may, at least partially, explain why different pathway choice strategies may have evolved in *T. gondii* in comparison with mammals.

The generation of a 5′ long end resected DNA, which prevents NHEJ process, is the key event that commits DSBs to HRR (Daley et al., 2013; Daley and Sung, 2014). The end resection requires different exonucleases (Exo1 and DNA2), the exo- and endonuclease Mre11, endonucleases (CtIP/Sae2), and helicases (BLM/Sgs1) (Figures S1, S2). Almost all the above-mentioned nucleases are positively regulated by cyclin dependent kinases (CDKs) mediated phosphorylations during G2/S-phase (Huertas et al., 2008; Huertas and Jackson, 2009; Ferretti et al., 2013). These data consolidate CtIP as a key player which redirects DSBs into HRR (Kakarougkas and Jeggo, 2014). In fact, phosphorylated CtIP cooperate with MRN (MRX in yeast) to facilitate end resection (Lafrance-Vanasse et al., 2015), reducing the chances for NHEJ activation. In mammals, phosphorylated CtIP also favors HRR activation by recruiting the mediator tumor suppressor protein breast cancer 1 (BRCA1) which is a BRCA1 C-terminal (BRCT) domain containing protein. BRCA1 evicts another BRCT-containing mediator, the NHEJ factor 53BP1 from the DSB (Daley and Sung, 2014). Intriguingly, 53BP1 also has a BRCT domain but, in opposition to BRCA1, 53BP1 function is that of inhibiting end resection (Chapman et al., 2012). In fact, 53BP1 binds canonical double-ended DSBs upstream the Ku heterodimer loading during G1, facilitating NHEJ (Bothmer et al., 2010). Interestingly, at one-ended DSBs the depletion of BRCA1 suffices to promote NHEJ while the simultaneous depletion of BRCA1 and 53BP1 restores HRR, therefore demonstrating an exquisite cross-regulation of HRR or NHEJ at DSBs (Bunting et al., 2010).

The lack of conservation in the above mentioned pathway choice step, that we postulate may happen in *T. gondii*, is intriguing. In fact, in yeast, only one BRCT-containing protein domain associated to HRR was found: Rad9 in *Saccharomyces cerevisiae* or Crb2 in *Schizosaccharomyces pombe*. The conservation between Rad9/Crb2 and mammalian BRCT containing proteins such as 53BP1 or BRCA1 is very low. Nevertheless, Rad9/Crb2 and 53BP1 functionally overlap. Similarly to 53BP1, Rad9 blocks end resection, and inhibits Exo1- and RAD50-dependent nucleases therefore inhibiting the formation of HRR-proficient substrates (Lazzaro et al., 2008). In concordance, NHEJ is facilitated when Rad9 is recruited to DSBs by the 9-1-1 checkpoint clamp loader (Ngo and Lydall, 2015). In addition, Rad9 acts as an adaptor that favors the activation of checkpoint kinases Mec1 (ATR) or Tel1 (ATM) and RAD53 and Chk1, which in turn facilitates successful finalization of S phase and promotes cell cycle arrest in G2 and G1, creating a time window for replication-dissociated DNA repair (Gilbert et al., 2001; Blankley and Lydall, 2004; Sweeney et al., 2005). Hence, the pathway choice in yeast may be tilted toward the choice of NHEJ, as we predict it may happen also in *T. gondii*.

Remarkably, according to the *T. gondii* database, homologs of BRCA1, 53BP1, or Rad9/Crb2 were so far not reported. We speculate that it is unlikely that such regulatory factors are completely missing in *T. gondii*. As the degree of conservation is low between yeast and mammals, it is possible that similar diversification may have taken place in *T. gondii*. In fact, similar functions were showed to be accomplished by different BRCT domains-containing proteins that share only few residues including hydrophobic amino acids which may facilitate the generation of appropriate secondary structure (Bork et al., 1997; Gabrielse et al., 2006). Interestingly, in *Toxoplasma* database are at least three putatives BRCT domain-containing-protein (Table 1). More work is required to establish whether such proteins are functional during the DSB pathway choice. Remarkably as well, homologous of CtIP and Nbs1 (Sae2 and Xrs2 in yeast, respectively) were also not present in *T. gondii* database (Table 1). While, Mre11 and RAD50 are conserved in all three domains of life and therefore also in *T. gondii* (Blackwood et al., 2013), the existence of a functional complex lacking Nsb1 is unconvincing to us. As it was already mentioned in Section DSB Recognition, the absence of Nbs1/Xrs2 annotation in *Toxoplasma* database, could be explained by the fact that this protein represents a highly divergent component of the MRN complex. Hence, in order to solve the molecular bases for the apparent defect in HRR activation in *T. gondii*, missing components need to be identified or their absence needs to be actively proved. In any case, the strong diversification of the HRR pathway that may have taken place in *T. gondii* may provide initial mechanistic bases for the predominant role of NHEJ in the repair of DSBs in the tachyzoite. We still believe that such conclusion may be precipitous since it is still unclear if different sets of proteins associated with the DSBs repair pathway choice has diversified in *T. gondii*. This is why in our opinion, the identification of mediators that rule the DSB pathway choice in *T. gondii* is a field that deserves much attention. Future work may ultimately address the role in DDR of TGME49_258480, TGME49_239790, and TGME49_237480, the three putative BRCT domain containing proteins. The search and identification of other putative BRCT containing proteins may also serve to comprehend HRR activation in *T. gondii* and to identify species-specific druggable targets for the treatment of toxoplamosis.

## γH2A.X spreading and foci formation

While H2A.X may be incorporated randomly in the genome of resting cells, its phosphorylated form γH2A.X, which is modified at its C-terminal motif SQEF/Y, can accumulate in discrete subnuclear foci at replication factories. γH2A.X is directly recruited to the site of DSBs or collapsed replication forks, a complex signaling network promotes the spreading of the γH2A.X signal along the chromosome from the damaged site up to 2-Mb (Redon et al., 2002). Such an increase in γH2A.X has also been reported when replication forks collapse in cells undergoing fast replication, such as precancerous and cancerous cells, showing an 8-fold increase both in levels of H2A.X and in γH2A.X when compared to resting cells (Bartkova et al., 2005). *T. gondii* tachyzoites also undergo fast DNA duplication and, similarly to cancer cells, increase γH2A.X as revealed by Western blot and mass spectrometry analysis (Dalmasso et al., 2009; Nardelli et al., 2013). The phosphorylation of the SQE motif of H2A.X in response to DSB relies on PIKK4 kinases ATM, ATR, or DNA-PK (van Attikum and Gasser, 2009), all of them apparently present in *T. gondii* (ATM and ATR are shown in Table S1). Intriguingly, while H2A.X is conserved in *T. gondii*, it is not present in all apicomplexas as well as other protozoan organisms (Dalmasso et al., 2009). This may suggest that the spreading of γH2A.X and foci formation in response of DSBs, while conserved in *T. gondii*, is not essential for HRR activation in all species.

The function of γH2A.X at DSB site and its spreading to both side of the DSB has been associated with the facilitation of homology search (Renkawitz et al., 2013) and with the recruitment of different components of DDR at the foci (Figures S1, S2). In mammals, one of the proteins that is recruited by γH2A.X is the BRCT-containing sensor MDC1 (mediator of DNA damage checkpoint protein 1), which was initially identified as a positive regulator of cell-cycle checkpoints effectors SMC1 and Chk1 during the S-phase and G2/M phases of the cell cycle (Stewart et al., 2003; Scully and Xie, 2013). MDC1 phosphorylation at its N-terminal region by casein kinase 2 (CK2) increases its interaction with Nbs1, enhancing the recruitment of the MRN complex at DSB site (Stewart et al., 2003; Melander et al., 2008; Spycher et al., 2008; Wu et al., 2008). Similarly, in yeast the checkpoint mediator Rad9/Crb2 relies on its C-terminal BRCT domain to interact with γH2A.X and to be recruited to the DSB (van Attikum and Gasser, 2009). In opposition to mammals, yeast has only one BRCT containing protein and therefore it is likely that a lower eukaryote as *T. gondii* may also have few BRCT-containing protein with HRR-regulating abilities. As mentioned above, *T. gondii* has three putative BRCT domain containing protein (TGME49_258480TGME49_239790 and TGME49_237480). In the future it will be important to determine whether these proteins have a function during DNA damage response and if that is the case, whether they act as mediator and/or has the ability to interact with γH2A.X.

## RAD52- independent HR pathway

Once the DSB is commited to HRR, the ssDNA generated by nucleases is immediately protected by RPA/RFA proteins. An important step is then the removal of RPA/RFA to allow the binding of the homology searching RAD51 recombinase to ssDNA. In Figures S1, S2 we summarize the current understanding of the mechanisms that regulate RAD51 loading to DNA (Zhang et al., 2009; Buisson et al., 2010, 2014; Dray et al., 2010; Ramadan, 2012; Mermershtain and Glover, 2013; Park et al., 2014). RAD52 is crucial both for displacement of RPA by RAD51 and for the stimulation of RAD51-mediated homologous DNA pairing (Baumann and West, 1999; Jackson et al., 2002). In *S. cerevisiae*, RAD52 plays a key role in HRR, but in vertebrates, RAD52 knockouts only have reduced HRR but do not have hypersensitivity to agents that induce DSBs (Rijkers et al., 1998; Paques and Haber, 1999). However, in vertebrates, the absence of both BRCA2 and RAD52 is synthetic lethal and is associated with severe chromosomal fragility (Feng et al., 2011). Hence, RAD52 and BRCA2 represent alternative pathways that converge to support RAD51-mediated HRR (Liu and Heyer, 2011; Lok and Powell, 2012). Moreover, BRCA2 displaces RPA from ssDNA and promotes RAD51 filament formation and strand exchange more efficiently than yeast and human RAD52 (Jensen et al., 2010). To this date it is unclear if *Caenorhabditis elegans* and *Drosophila melanogaster* have a RAD52 homolog but they do have a BRCA2 protein (Liu and Heyer, 2011). In *C. elegans*, BRCA2 (CeBRC-2) stimulates both RAD51-mediated D-loop formation and single strand annealing of RPA-oligonucleotide complexes (Petalcorin et al., 2006). This suggests that CeBRC-2 may have taken over the role of vertebrate RAD52 in DNA single-strand annealing.

As for other mediators, we and others have found no evidence of RAD52 expression in *T. gondii*, Plasmodium spp., and trypanosomatids genome (Passos-Silva et al., 2010; Lee et al., 2014; Smolarz et al., 2014). In contrast, RAD52 has been identified in *Entamoeba hystolytica* and *Giardia* spp. (Lopez-Camarillo et al., 2009). Hence, it is possible that RAD52 may indeed not be part of the DNA damage response pathway in *T. gondii* and protozoan parasites. In such scenario, the recruitment of *T. gondii* RAD51 to the DSB might be controlled by a RAD52-independent mechanism as proposed Smolarz et al. (2014). Interestingly, it is also possible that the putative BRCA2 may represent the sole protein in charge of displacing RPA and recruiting RAD51 to ssDNA in this organisms, a scenario which is not exceptional (Petalcorin et al., 2006; Liu and Heyer, 2011).

BRCA2 is a protein with multiple domains, including oligonucleotide/oligosaccharide-binding (OB) domains, BRC tandem repeats, and TR2 C-terminal domain. In human BRCA2, the three OB repeats are implicated on ssDNA binding, whereas the BRC repeats promote the protein-protein interactions that facilitate the DNA binding and the focal organization of RAD51(Flynn and Zou, 2010). Moreover, the TR2 domain in the BRCA2 C-terminus stabilizes RAD51 nucleoprotein filament (Lee, 2014). Depending on the organisms, the interaction of BRC domains with RAD51 can be weak or strong therefore positively or negatively impacting on the control over RAD51's activities (Davies et al., 2001). For the putative BRCA2 protein, TGME49_243265, that we have found in *T. gondii* database (Table 1), it could be identified two BRCA2 domains. One at position 2505-2619 (pfam09103) and other at position 760 to 1838. Hence, near 16 repeat sequences in TGME49_243265 presents striking similarities to the BRC repeats present in humans or *Trypanosoma brucei* (Trenaman et al., 2013; Lee, 2014). Interestingly, in *T. brucei*, RAD51 encodes a high number of BRC repeats which facilitate RAD51 foci formation. As the number of cells with detectable RAD51 foci is proportional to the number of BRC-repeats (Trenaman et al., 2013), the increased BRC repeats in RAD51, might be relevant when attempting RAD51 loading in the absence of multiple mediators.

In mammals, ubiquitylated H2A.X recruits a complex of proteins which promote BRCA2 loading to DNA (Scully and Xie, 2013). Hence, the analysis of post-translational modifications in histones of *T. gondii* may be informative. Recent reports indicates that this organism has four detectable ubiquitylation on H2A.X (Silmon de Monerri et al., 2015). It will be of interest to determine the role of these PTMs on *T. gondii* H2A.X in HRR.

## The role of chromatin in HRR

In addition to H2A.X, several PTMs of histones such as acetylation, phosphorylation, methylation, and ubiquitination, occur at regions of damaged DNA(Gospodinov and Herceg, 2013). Moreover, chromatin-remodeling complexes INO80, SWR1, SWI/SNF, RSC, and NuRD are all important initiators of the DSB repair pathway in both low and high eukaryotes (van Attikum and Gasser, 2009). Likewise, H2A.Z may have a crucial role in defining the extent of the nucleosome-free DNA regions, restricting DNA resection by CtIP and favoring the recruitment of NHEJ initiators such as the Ku70/Ku80 complex (Xu et al., 2012). The remodeling of chromatin not only facilitates DNA repair but also prevents stalled forks from collapsing and promotes their subsequent restart (Vassileva et al., 2014).

Histone acetylations are generated by histone acetyl transferases (HAT) as the members of the MYST (e.g., Tip60, Esa1) or GCN5 family (Gardner et al., 2011). NuA4-Tip60 complex participates in two major DDR steps which are the remodeling of chromatin at DSBs and the acetylation and activation of the ATM kinase (Sun et al., 2010). The Tip60 chromodomain interacts with H3 trimethylated on lysine 9 (H3K9me3) at DSB and then the NuA4-Tip60 complex acetylates H4 to generate H4K16Ac (Kusch et al., 2004; Daley and Sung, 2014). Once recruited to chromatin, Tip60 also acetylates ATM in the proximity of DSB, facilitating the phosphorylation of several HRR proteins required to achieve efficient HRR (Sun et al., 2007) (Figure S1 and Figure 2). At the same time, the acetylation of lysine 16 on H4 (H4K16Ac) disfavors the recruitment of 53BP1 to H4K20me and prompts HRR by enhancing the loading of BRCA1 to DSB (Tang et al., 2013). A recent report showed that H3K56Ac is important for the activation of a HRR-dependent events known as sister chromatid exchange (Munoz-Galvan et al., 2013).

As mentioned above, H3K9me is crucial to recruit a NuA4-Tip60 complex to DSBs. Defective H3K9 methylation negatively regulates HRR favoring NHEJ (Ayrapetov et al., 2014). Other methylations of histones such as H3K79me and H4K20me2 are also relevant for NHEJ-directed DSB repair, potentially acting as docking sites for the recruitment of DNA repair factors including 53BP1 (Hsiao and Mizzen, 2013). In yeast, Set2-dependent H3K36 methylation (H3K36me) reduces the chromatin accessibility of HRR factors and the resection of DNA-ends promoting NHEJ. In contrast, GCN5-dependent H3K36 acetylation promotes HRR by increasing the chromatin accessibility to HRR factors and the resection of DNA-ends (Pai et al., 2014). As mentioned in previous sections, histone ubiquitination, in particular histone H2A, is another PTM relevant for the recruitment of different HRR associated proteins to DSBs (see above and Figure S1).

In *T. gondii*, MYST-B were proposed to function as putative TIP60 HATs (Vonlaufen et al., 2010). Furthermore, two GCN5 (isoforms A and B) were also reported in the parasite (Sullivan and Hakimi, 2006). Histones H2A.Z, H2A.X and a novel histone variant H2B.Z, which forms a novel nucleosome integrating a double variant of H2A.Z/H2B.Z were also described in *T. gondii* (Dalmasso et al., 2009). These observations suggest the conservation in *T. gondii* of the chromatin remodeling factors required during the onset of DSB repair. As mentioned above, overexpression of tagged TgMYST-B reduces growth rate *in vitro* and confers protection from the methyl methanesulfonate DNA-alkylating agent (Vonlaufen et al., 2010). These results suggest a role of this HAT in the activation of DNA repair and/or in the prevention of fork collapse. Despite the high homology between the HAT domains, the two TgGCN5s exhibit differential substrate specificities. While TgGCN5-A exclusively targets lysine 18 of H3 (H3K18), TgGCN5-B acetylates multiple lysines in the H3 tail (Bhatti et al., 2006). TgGCN5-A is dispensable for the proliferation of the parasite *in vitro*, but it is required for the parasite recovery when challenged with alkaline stress (Naguleswaran et al., 2010). In contrast, the expression of a catalytically inactive TgGCN5-B arrests the cell outside S-phase (Wang et al., 2014). Despite the initial evidences discussed above, further investigation is required to determine whether one or both TgGCN5 participate in HRR.

Interestingly, some of the DSB-triggered histone's PTMs are conserved in *T. gondii*. The phosphorylation of the SQE motif in TgH2A.X was observed in RH strain treated with oxidative stress agents such as H_2_O_2_ (Dalmasso et al., 2009). Other conserved PTMs in *T. gondii* include HRR- (H4K16Ac and H3K9me) and NHEJ-associated marks (H3K36me, H3K79me, and H4K20me2) (Nardelli et al., 2013) *T. gondii* H4K16me3 was also detected, suggesting a putative regulation of this mark by signals arising from the accumulation of damaged DNA (Nardelli et al., 2013). To note, the PTM map of *T. gondii* histones was generated from tachyzoites samples grown in unperturbed conditions. In light of these facts, it can be proposed that in *T.gondii* the remodeling of chromatin and the changes in histones PTMs may also participate in the choice between NHEJ- and HRR-directed repair of DSBs. However, clear differences with other species such as the undetectable acetylation of histone H3 at K3 and 36 were also revealed. Moreover, the identification of a novel H2B.Z isoform specific for *T. gondii*, confirms a certain level of diversification with other species. We believe that the biological relevance of such differential regulation should be promptly explored as it can provide tools for the design of specific treatments that impair DSBs repair in *T. gondii* but not in its host.

## Conclusions and future perspectives for novel drug targets

This review has discussed the multiple evidences that support the conservation of pathways in charge of DSB repair such as HRR in *T. gondii* and its parent *Plasmodium* spp. Since HRR requires sister chromatids as a template for DNA repair we reasoned that the highly proliferative stages of *T. gondii* would highly depend on HRR after DSB accumulation. The tachyzoite stage is characterized by the highest replication rate in T. *gondii*, while the bradyzoite replicates within the cyst *in vivo* (Watts et al., 2015). It is therefore expected that at least in the tachyzoite, HRR would be the preferred pathway choice. However, the insertion of DSB-like plasmid suggests that NHEJ is the preferential mechanism of DSBs in tachyzoites while HRR events are evidenced only if NHEJ is blocked by elimination of *Ku80* (Fox et al., 2009). Whether NHEJ is always the preferred pathway chosen under all conditions of DSB generation remains to be tested. We anticipate that this is highly unlikely, at least for single-ended DSBs at collapsed forks, as the repair of such lesions by the NHEJ pathway should cause lethal chromosomal fusions.

To improve the understanding of the DSB repair pathway choice in *T.gondii*, the identification of all parasite factors regulating such decision is required. Our analysis suggests that the basic components of the HRR machinery are conserved in *T. gondii*. However, the picture is incomplete particularly when focusing on the factors in charge of the choice between HRR/NHEJ (mediators, CtIP, Nbs1, EME1, etc). Similar limitations were reported in *Plasmodium* spp. (Table S1), an organism that clearly chooses HRR in many instances such as the generation of var gene antigen in the subtelomeric gene family and the sequence diversification and *var* gene family composition during mitosis (Lee et al., 2014). Hence, it is possible that, by identifying key mediators in *T.gondii* and *Plasmodium* spp., specific druggable factors useful for the treatment of toxoplasmosis may be revealed.

Another important subject that requires further investigation is the evaluation of the extent of DSB accumulation and HRR activation during the tachyzoite stage. High rates of DNA replication may increase the rates of replication forks collapse generating one-ended DSB which require the HRR for fork restart and cell survival (Lee et al., 2014). In that context, HRR might be essential for the repair of collapsed forks, in particularly after treatment with anti-tumoral compounds such as topoisomerase I inhibitors including Camptothecin (CPT), irinotecan, topotecan (Tomicic and Kaina, 2013). Interestingly, the combination of treatments that increase one-ended DSB with an HRR defect emerged as a novel, potent and synthetic lethal alternative for cancer treatment (Batey et al., 2013). Moreover, it has been recently suggested that HRR regulators can represent suitable candidates for anti-cancer therapy (Batey et al., 2013; Krajewska et al., 2015). In fact, there are some drugs that target factors such as the MRN/X complex or the ATM kinase that show synergism when used with DNA damage drugs such as cisplatin and PARP inhibitors including Olaparib. Moreover, a repertoire of small and microRNAs and peptides that target different HRR proteins such as BRCA1, BRCA2, RAD51, BLM among others, also cause synthetic lethal effects when combined with PARP inhibitors (Farmer et al., 2005). We postulate that the knowledge of the structural basis of protein-protein interactions required for HRR activation (Mermershtain and Glover, 2013) may serve to design small molecule inhibitors specific for the DSB repair pathway in *T.gondii*. We therefore consider that it is crucial to promptly identify the missing components of the HRR pathway in *T. gondii*. If factors that have diversified from humans are validated they may represent a unique source of druggable targets, which could be used along with clasical DNA damaging agents to improve current anti-toxoplasmic therapies.

Last but not least, HRR independent functions of BRCA2, RAD51 and the exonucleases Mre11, DNA2 were recently reported. These factors can initiate and/or regulate the extent of exonucleolytic cleavage of nascent DNA in conditions of persistent stalling of replication forks (Schlacher et al., 2011; Thangavel et al., 2015). Such events are independent from DSBs formation and other HRR factors such as RAD54. The HRR-indpendent function of the above-mentioned factors is required to protect the genomic stability of human cells. Therefore, it will be important to evaluate if there is a HRR-independent contribution of the putative BRCT-containing proteins and the RAD51 of *T. gondii* in the protection of stalled forks. The evaluation of the level of conservation of such cascade and the evaluation of its contribution to the genomic stability of the parasite will be very important when attempting to design specific targeted theapies for the treatment of toxoplasmosis.

## Author contributions

All of the authors reviewed the data from the literature and organized and wrote the manuscript. IF, SC, and SA were involved in design the figures. IF, VG, and SA were involved in editing the final version of the manuscript. All of the authors read and approved the final version of the manuscript.

### Conflict of interest statement

The authors declare that the research was conducted in the absence of any commercial or financial relationships that could be construed as a potential conflict of interest.
