# Supplementary material for: The Knowns Unknowns: Exploring the Homologous Recombination Repair Pathway in Toxoplasma gondii
Source: Front Microbiol. 2016 May 3;7:627. doi: 10.3389/fmicb.2016.00627 (PMC4853372; doi:10.3389/fmicb.2016.00627)
Supplement: Supplementary file 1 [file DataSheet1.pdf]

Supplementary Material

Known Homologous recombination repair pathway unknown in the *Toxoplasma gondii*

Ignacio M. Fenoy[1], Vanesa Gottifredi[2], Silvina S. Bogado[1], Marisol S. Contreras[1], Sergio O. Angel[1],*

[1]Laboratorio de Parasitología Molecular, IIB-INTECH, CONICET-UNSAM, Argentina

[2] Cell Cycle Genomic Instability Laboratory, Fundación Instituto Leloir, IIBBA-CONICET, Buenos, Aires, Argentina

***Correspondence:** Sergio Angel. Laboratorio de Parasitología Molecular, IIB-INTECH, CONICET-UNSAM, Av. Intendente Marino Km. 8.2, C.C 164, (B7130IIWA), Chascomús, Prov. Buenos Aires, Argentina. Tel. +54 2241 430323; e-mail: sangel@intech.gov.ar

1 Supplementary Figures

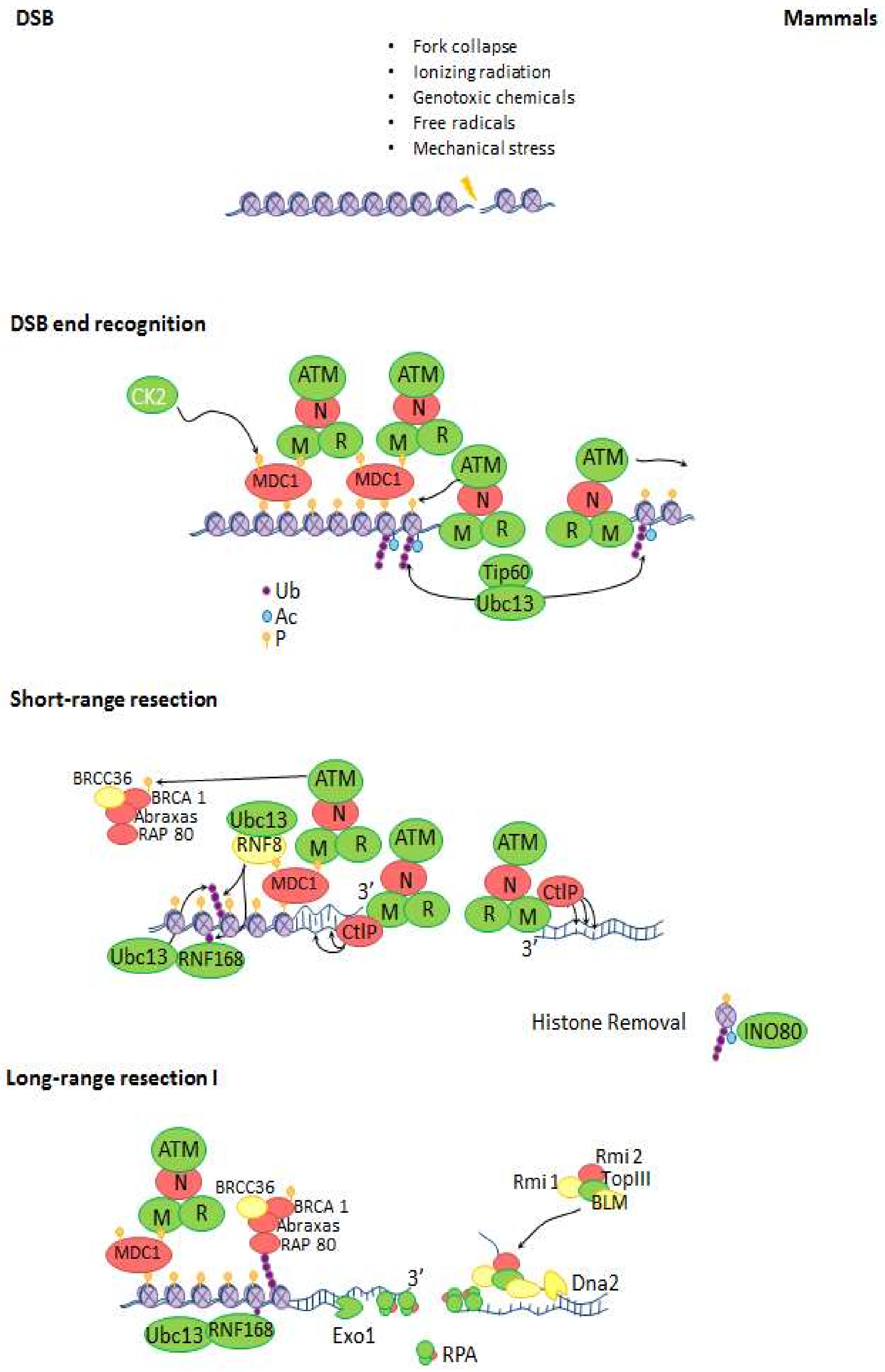

DSB
Mammals
Fork collapse
Ionizing radiation
Genotoxic chemicals
Free radicals
Mechanical stress
DSB end recognition
CK2
ATM
N
M
R
MDC1
Tip60
Ubc13
Ub
Ac
P
Short-range resection
BRCC36
BRCA 1
Abraxas
RAP 80
RNF8
RNF168
CtIP
3'
Histone Removal
INO80
Long-range resection I
Rmi 2
Rmi 1
TopIII
BLM
Dna2
Exo1
RPA

Supplementary Figure 1: Homologous recombination in Mammals. After DSB, DNA end resection depends on the Mre11-RAD50-Nbs1 (MRN) sensor complex, the exonuclease EXO1 and the phosphorylated form of the exonuclease CtIP. First, DNA ends are bound by MRN. Mre11 possesses single-strand endonuclease activity and double-strand-specific 3'-5' exonuclease activity, RAD50 may be required to bind DNA ends and hold them in close proximity. Nbs1 is formed by different domains: Forkhead associated domain (FHA); BRCT domain, Mre11-binding domain and C-terminal protein interaction motif as well as two ATM-phosphorylation sites. Nbs1 interacts with Mre11/RAD50 in focal structures which surround the DSB and which are required for optimal activation of the PI3Kinase, ATM. In turn, ATM activation phosphorylates checkpoint kinases, (Difilippantonio and Nussenzweig, 2007) histone H2A.X, MDC1 and BRCA1 proteins. The association of BRCA1 adaptor/mediator with CtIP enhances the nuclease activity of the MRN complex which is essential for the initiation of the nucleolytic resection of DSBs Phosphorylated H2A.X on SQE motif (γH2A.X) recruit phosphorylated MDC1 which in turn recruits other MRN complexes. The latter complex, initiates a second round of recruitment of ATM, which thereafter triggers the spreading of the DNA damage response at DSB flanking regions. γH2A.X also recruits INO80 which mediates histones eviction. MRN slides 100-200 base pairs away from the break and recruits CtIP which stimulates the endonuclease activity of Mre11, promoting an endonucleolytic incision which is followed by an MRE11-dependent 3′-5′exonuclease digestion towards the DNA end. Such MRE11-dependent processing of the DSB is coupled with an EXO1/BLM-dependent 5′-3′ resection away from the end (Shibata and Jeggo, 2014) (Lafrance-Vanasse et al., 2015). The ssDNA formed is rapidly stabilized by RPA (Symington and Gautier, 2011). RNF8 forms a complex with Ubc13, an E2 ubiquitin conjugating enzyme, and catalyzes the 'Lys-63'-linked ubiquitination of histones H2A and H2A.X, thereby promoting the formation of BRCA1 foci. Interestingly, RNF8 also catalyzes 'Lys-63' ubiquitination that targets proteins at DSB to the proteasome (Ramadan, 2012).

After that, RNF168, another E3 ubiquitin ligase is recruited by Ubc13. The ubiquitylation of H2A/H2B in 'Lys-63' recruits RAP80-Abraxas-BRcc36 complex to the DSBs repair foci thorough RAP80-ubiquitin interacting motifs (UIM). The latter factor recruits BRCA1-BARD1 heterodimer which thereby results in the recruitment of PALB2/FANCN and subsequently BRCA2/FANCND1 at foci (Zhang et al., 2009;Mermershtain and Glover, 2013). BARD1 is not shown in the figure. The ability of PALB2 to form homo-oligomers facilitates its interaction with BRCA2, RAD51 and pol η, promoting formation of a D-loop during strand invasion recombination-associated DNA synthesis (Buisson et al., 2010;Dray et al., 2010;Buisson et al., 2014). Proteins with high level of conservation between *T.gondii* and humans are colored in green. Proteins with medium or low level of conservation are colored in yellow and those which were not identified in *T. gondii* are colored in red.

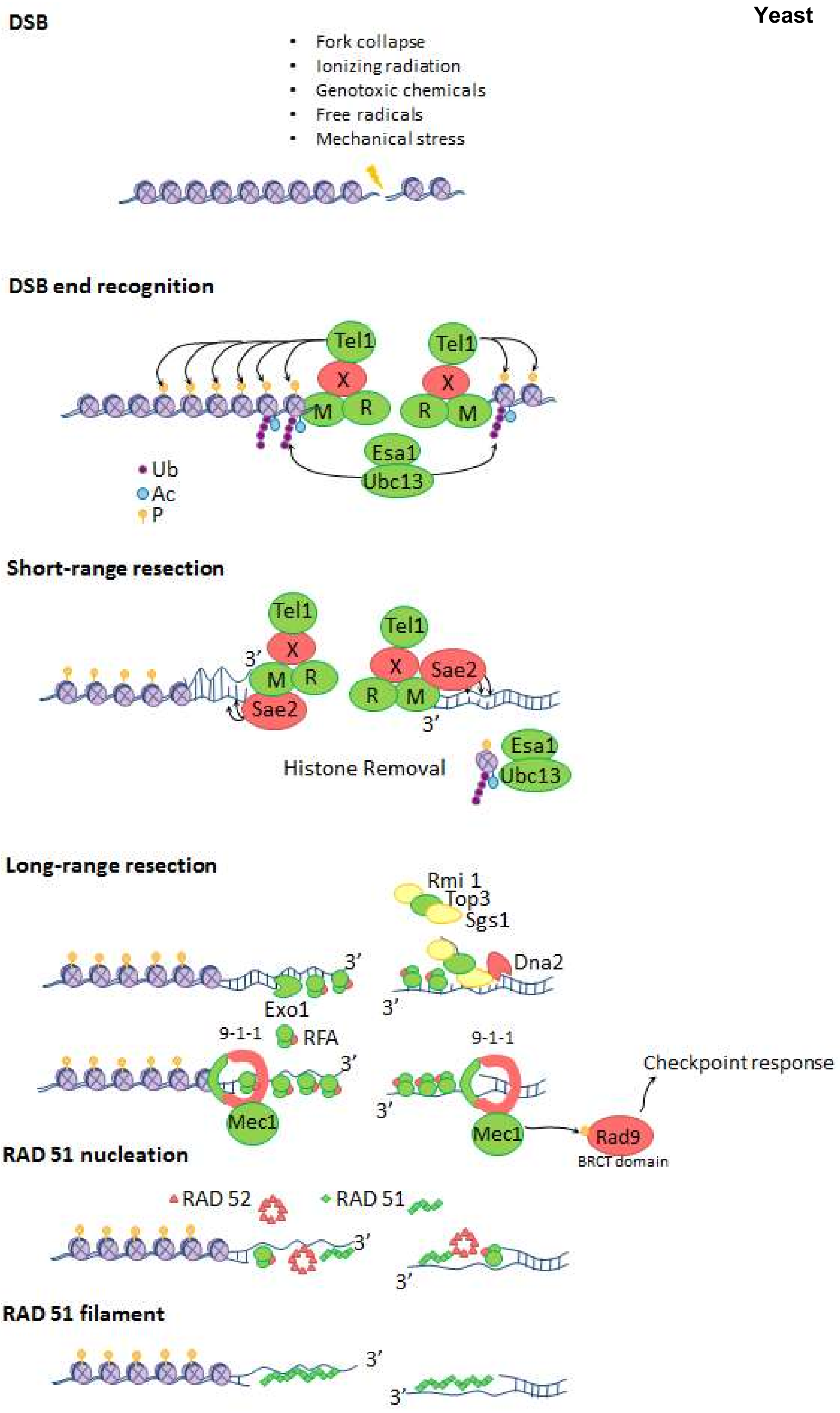
DSB
Yeast
Fork collapse
Ionizing radiation
Genotoxic chemicals
Free radicals
Mechanical stress
DSB end recognition
Tel1
X
M
R
Tel1
X
R
M
Esa1
Ubc13
Ub
Ac
P
Short-range resection
Tel1
X
3'
M
R
Sae2
Tel1
X
Sae2
R
M
3'
Histone Removal
Esa1
Ubc13
Long-range resection
Rmi 1
Top3
Sgs1
3'
Dna2
Exo1
3'
9-1-1
RFA
3'
9-1-1
Checkpoint response
3'
Mec1
Mec1
Rad9
BRCT domain
RAD 51 nucleation
RAD 52
RAD 51
3'
3'
RAD 51 filament
3'
3'

Supplementary Figure 2: Homologous recombination in yeast. Similar to mammals, the end resection of DNA DSB depends on Mre11-RAD50-Xrs2 (MRX) sensor complex, the nucleases EXO1 and Sae2 proteins. Phosphorylated Xrs2 recruit the PIK3 Tel1 kinase which phosphorylates H2A.X to spread γH2A.X at homologous recombination foci (Lisby et al., 2004) (Redon et al., 2003). Next the BRCT domains containing Rad9/Crb2 adaptor binds chromatin flanking the DSB sites and the 5'-3'end resection is achieved by Sae2. The inactive form of Sae2 forms large oligomeric structures which upon CDK phosphorylation at Ser267 during S/G2 –phase are dissociated into monomeric and dimeric active forms, becoming a key factor in the choice of HR process (Fu et al., 2014). Sae2 together with Exo1 and the helicase–endonuclease Sgs1–Top3–Rmi1 (STR)–Dna2 complex (BLM–TopoIIIα–RMI1/RMI2 (BTR)–DNA2 complex12) generate a long ssDNA that is coated by Rfa 1-3 proteins.

The ssDNA /Rfa1-3 complexes recruit the PCNA-like 9-1-1 complex as well as the PIKK Mec1 (ATR in mammals)(Lisby et al., 2004). Mec1 phosphorylates Rad9/Crb2 which binds RAD53 (Chk2 in mammals) coupling this effector in DNA damage checkpoint (Schwartz et al., 2002). On the other hand, the mediator RAD52 promotes the displacement of RFA from ssDNA and the formation of a RAD51 nucleoprotein filament (Sung, 1997). The histone acetyltransferase (HAT)nd Ubc13 modify the histones post-transcriptionally allowing their eviction from the damaged-DNA region. Proteins with high level of conservation between *T.gondii* and yeast are colored in green. Proteins with medium or low level of conservation are colored in yellow and factors wich have not been yet identified in in *T. gondii* are colored in red.

2. Supplementary Table

Supplementary Table S1: List of HRR protein in human, *Saccharomyces cerevisiae*, *T. gondii* and *Plasmodium falciparum*. The description was obtained from UNIPROT database (human and yeast), www.toxodb.org (*T. gondii*) and www.plasmodb.org (*P. falciparum*). *T. gondii* and *P. falciparum* HRR sequences were retrieved by Blastp using human and yeast amino acidic sequence. In some cases, *T. gondii* putative HRR proteins sequences were used to find the counterpart in *P. falciparum* and vice versa. ND: not detected, -: not present. In Blue letter: BRCT domain containing proteins.

H. sapiens	UNIPROT	S. cerevisiae	UNIPROT	alias/function	T. gondii	description	P. falciparum	description
Abraxas	Q6UWZ7	_	_	BRCA1-A complex subunit Abraxas;	ND	_	ND	_
ATM	Q13315	Tel1	P38110	PI3_4_KINASE	TGME49_248530	FATC domain-containing protein	PF3D7_0515300	phosphatidylinositol 3-kinase (PI3K)
ATR	Q13535	Mec1	P38111	PI3_4_KINASE	TGME49_283702	FATC domain-containing protein	PF3D7_0515300	phosphatidylinositol 3-kinase
Bard1	Q99728	_	_	BRCA1-associated RING domain protein 1	ND	_	ND	_
BLM	P5413	Sgs1	P3518	RecQ ATP-	ND	_	ND	_

	2		7	dependent helicase				
Brca1 C-terminus=								
53BP1	Q12888	_	_	TP53BP1; p53-binding protein 1; p202;	ND	_	ND	_
BRCA1	P38398	_	_	Breast cancer type 1	ND	_	ND	_
MDC1	Q14676			Mediator of DNA damage checkpoint	ND	_	ND	_
_	_	RAD9	P14737	DNA repair protein RAD9	ND	_	ND	_
_	_	_	_	_	TGME49_239790	BRCT containing protein	_	_
_	_	_	_	_	TGME49_237480	BRCT containing protein	_	_
_	_	_	_	_	TGME49_258480	hypothetical protein	ND	_
BRCA2 (FANCD1)	P51587	_	_	Breast cancer type 2	TGME49_243265	putativa BRCA2, protamine P1 protein	PF3D7_1328200	conserved Plasmodium protein, unknown function

BRCC36	P46736	_	_	lys-63-specific deubiquitinase	TGME49_308590	Mov34/MPN/PAD-1 family protein	PF3D7_1368100	26S proteasome regulatory subunit RPN11
CDC25A	P30304	YCH1	P42937	M-phase inducer phosphatase 1	ND	_	ND	_
ChK1	O14757	ChK1	P38147	serine/threonine-protein kinase ChK1	ND	_	ND	_
ChK2	O96017	Rad53	P22216	Serine/threonine-protein kinase Chk2	TGME49_207820	cell-cycle-associated protein kinase MAPK	PF11_0147	mitogen-activated protein kinase 2 (MAP2)
CK2 alpha	P68400	CKA2	P19454	Catalytic subunit of a constitutively active serine/threonine-protein kinase complex	TGME49_263070	CMGC kinase, CK2 family	PF3D7_1108400	casein kinase 2, alpha subunit
CK2 beta	P67870	CKB1	P43639	CK2 regulatory subunit	TGME49_272400	casein kinase ii regulatory subunit protein	PF3D7_1103700	casein kinase II beta chain
CtIP	Q99708	Sae2	P46946	DNA endonuclease	ND	_	ND	_
DMC1	Q14565	DMC1	P25453	Meiotic recombination protein	TGME49_216400	meiotic recombination protein	PF3D7_0816800	meiotic recombination protein

				DMC1		DMC1 family protein		DMC1, putative (DMC1)
DNA polymerase h	Q7Z5Q5	–	–	translesion synthesis	TGME49_237830	DNA polymerase I domain-containing protein	PF3D7_1411400	plastid replication-repair enzyme (PREX)
DNA polymerase δ	P28340	DNA polymerase δ	P46588	DNA polymerase delta catalytic subunit	TGME49_258030	DNA polymerase , catalytic subunit	PF3D7_1017000	DNA polymerase delta catalytic subunit
DNA2	P51530	Dna2	P38859	DNA replication ATP-dependent helicase/nuclease	TGME49_269740	R3H domain-containing protein	PF3D7_1106700	dna2/nam7 helicase family member
EME1	Q96AY2	MMS4	P38257	Crossover junction endonuclease	TGME49_305310	ERCC4 domain-containing protein	PF3D7_1368800	DNA repair endonuclease
ERCC1	P07992	Rad10	P06838	DNA excision repair protein iosoform 1	TGME49_249330	DNA repair protein rad10 subfamily protein	PF3D7_0203300	ERCC1 nucleotide excision repair protein, putative
ERCC4 (XPF4)	Q92889	Rad1	P06777	DNA repair endonuclease XPF	TGME49_305310	ERCC4 domain-containing protein	PF3D7_1368800	DNA repair endonuclease
EXO1	Q9UQ84	Exo1	P39875	exonuclease 1	TGME49_233090	XPG N-terminal domain-containing	PF3D7_0725000	exonuclease I, putative

						protein		
FANCD2	Q9BXW9	_	_	Fanconi anemia group D2 protein	ND	_	_	_
FANCF	Q9NPI8			ubiquitin-protein transferase activity	ND	_	ND	_
FANCM	Q8IYD8	Mph1	P40562	ATPase required for FANCD2 ubiquitination	ND	_	ND	_
GCN5 (KAT2A)	Q92830	GCN5	Q03330	Histone acetyltransferase GCN5	TGME49_254555	histone lysine acetyltransferase GCN5-A	PF3D7_0823300	histone acetyltransferase GCN5
"	"	"	"	"	TGME49_243440	histone lysine acetyltransferase GCN5-B	"	"
GEN1 (FLAP)	Q17RS7	Yen1	P40028	Flap endonuclease GEN homolog 1	TGME49_251620	flap structure-specific endonuclease 1	PF3D7_0206000	DNA repair endonuclease, putative
H2A.X	P16104	HTA2	P04912	histone	TGME49_261580	histone H2A.X	ND	
Hop2	Q9P2W1	Hop2	P53187	Homologous-pairing protein 2	ND	_	ND	_
HP1 alpha(CBX5)	P45973	Swi6 (S. pombe)	P40381	Heterochromatin protein 1	TGME49_268280	chromo' (CHRromatin Organization	PF3D7_1220900	heterochromatin protein 1

						MOdifier)		
HUS1	O60921	Mec3	Q02574	Checkpoint protein	TGME49_206390	Hypotetical protein	ND	_
MOF (KAT8)	Q9H7Z6	–	–	histone acetyltransferase (MYST samily)	TGME49_318330	histone lysine acetyltransferase MYST-A	PF3D7_1118600	histone acetyltransferase, putative (MYST)
MRE11	P49959	Mre11	P32829	single-strand endonuclease activity and double-strand-specific 3'-5' exonuclease activity	TGME49_278060	Mre11 DNA-binding domain-containing protein	PF3D7_0107800	double-strand break repair protein MRE11
MUS81	Q96NY9	Mus81	Q04149	Crossover junction endonuclease	TGME49_261610	Hypotetical protein	PF3D7_1449400	DNA replication related protein
Nbs1	O60934	Xrs2	P33301	modulate the DNA damage signal	ND	_	ND	_
KU70(XRCC6)	P12956	Ku70	P32807	ATP-dependent DNA helicase II subunit 1	TGME49_248160	Hypotetical protein	ND	_
KU80(XRCC5)	P13010	Ku80	Q04437	ATP-dependent DNA helicase II subunit 2	TGME49_312510	Ku70/Ku80 beta-barrel domain-containing protein	ND	_

p53	P04637	_	_	Cellular tumor antigen p53	ND	_	ND	_
PALB2/FANCN	Q86YC2	_	_	recruit BRCA2 and RAD51 to DNA breaks	ND	_	ND	_
PCNA	P12004	PCNA (POL30)	P15873	control of eukaryotic DNA replication by increasing the polymerase's processibility during elongation of the leading strand	TGME49_247460	proliferating cell nuclear antigen 1	PF3D7_1361900	proliferating cell nuclear antigen
PCNA	"	"	"	"	TGME49_320110	proliferating cell nuclear antigen 2	PF3D7_1226600	proliferating cell nuclear antigen 2
RAD50	Q92878	Rad50	P12753	structural maintenance of chromosomes (SMC) family - Assembly of the RAD50-MRE11-NBS1 complex at DNA double-strand	TGME49_257180	RecF/RecN/SMC	PF3D7_0605800	DNA repair protein RAD50

				breaks				
RAD51	Q06609	Rad51	P25454	recombinase, binds to single and double-stranded DNA	TGME49_272900	DNA repair protein RAD51, putative	PF3D7_1107400	Rad51 homolog (RAD51)
RAD51B	O15315	–	–	DNA repair protein RAD51 homolog 2	ND	_	ND	–
RAD51C	O43502	–	–	DNA repair protein RAD51 homolog 3	ND	_	ND	–
RAD51D	O75771	–	–	DNA repair protein RAD51 homolog 4	ND	_	ND	–
RAD51AP1	Q96B01	–	–	RAD51-associated protein 1	ND		ND	–
RAD52	P43351			stimulation of the RAD51 recombinase	ND	_	ND	_
RAD54	Q92698			DEAD/DEAH helicase	TGME49_232450	SWI2/SNF2-containing protein RAD54	PF3D7_0803400	DNA repair protein RAD54
_	_	Rad55	P38953	RAD51 paralog	ND	_	ND	_

_	_	Rad57	P25301	RAD51 paralog	ND	_	ND	_
RAP80 (UIMC1)	Q96RL1	_	_	binds 'Lys-63'-linked ubiquitin	ND	_	ND	_
RMI1	Q9H9A7	Rmi1	Q02685	RecQ-mediated genome instability protein 1	ND	_	ND	_
RMI2	Q96E14	_	_	RecQ-mediated genome instability protein 2	ND	_	ND	_
RNF168	Q8IYW5	Rad18	P10862	histone H2A E3 ubiquitin ligase	ND	_	ND	_
RNF8	O76064	Dma2	P53924	histone H2A E3 ubiquitin ligase	ND	_	ND	_
RPA1	P27694	RFA1	P22336	binds and stabilizes single-stranded DNA intermediates	TGME49_236080	replication factor-a protein 1 (rpa1) subfamily protein	PF3D7_0409600	replication protein A1, large subunit (RPA1)
RPA2	P15927	RFA2	P26754	binds and stabilizes single-stranded DNA intermediates	ND	_	ND	_

RPA3	P35244	RFA3	A6ZQG0	binds and stabilizes single-stranded DNA intermediates	TGME49_214480	replication factor a protein 3 protein	PF3D7_1442100	replication factor A protein 3, putative
_	_	RSC2	Q06488	Chromatin structure-remodeling complex				
_	_	Srs2	P12954	ATP-dependent DNA helicase	TGME49_277550	UvrD/REP helicase domain-containing protein	PF3D7_0514100	ATP-dependent DNA helicase UvrD
SLX1	Q9P7M3	Slx1	P38324	structure-specific endonuclease	TGME49_212170	GIY-YIG catalytic domain-containing protein	PF3D7_1250800	DNA repair protein rhp16, putative
SLX4 (FANCP)	Q8IY92	Slx4	Q12098	Structure-specific endonuclease subunit	TGME49_277540	Hypotetical protein	ND	_
SMC1	P32908	Smc1	P32908	structural maintenance of chromosomes protein	TGME49_288700	RecF/RecN/SMC N terminal domain-containing protein	PF3D7_1318400	chromosome segregation protein
SMC3	Q9UQE7	Smc3	P47037	structural maintenance of chromosomes protein	TGME49_297800	RecF/RecN/SMC N terminal domain-containing protein	PF3D7_1318400	chromosome segregation protein

SPO11	Q9Y5K1	Spo11	P23179	Meiotic recombination protein	ND	_	ND	_
TOPOIII	Q13472	Top3	P13099	Releases the supercoiling and torsional tension of DNA	TGME49_264450	DNA topoisomerase III beta-1	PF3D7_1347100	DNA topoisomerase III
UBC13	O13685	Ubc13	P52490	Ubiquitin-conjugating enzyme E2	ND	_	ND	_
Wrn1	Q14191	_	_	Werner syndrome ATP-dependent helicase	TGME49_306080	ATP-dependent DNA helicase, RecQ family protein	PF3D7_1429900	ATP-dependent DNA helicase
XRCC2	O43543	_	_	DNA repair protein	ND	_	ND	_
XRCC3	O43542	_	_	DNA repair protein	ND	_	ND	_
